# Air Quality in a Changing Climate

**DOI:** 10.1289/ehp.1103649

**Published:** 2011-04

**Authors:** Dan Costa

**Affiliations:** Air, Climate and Energy, Office of Research and Development, Environmental Protection Agency, Research Triangle Park, North Carolina, E-mail: costa.dan@epa.gov

Once, in the not so distant past, smoke belching from urban factories and plants was viewed as a symbol of a prosperous, growing economy with abundant jobs. However, awareness of the downside of such pollution spawned the environmental movement of the 1960s, culminating in the Clean Air Act Amendment of 1970. This landmark legislation provided the regulatory mandates and legal tools that stimulated innovative technological efficiencies. Remarkably, over the next 40 years, emissions were brought down by more than two-thirds, even as productivity improved.

Today, the absence of sooty urban air and the presence of clear vistas are the visible hallmarks of successful air pollution policies. Indeed, in the past two decades, the percentage of people who view the threat of air pollution with “a great deal of concern” has been reduced by 25% (Jones 2010). Even the Office of Management and Budget (2007) noted that air pollution regulations have yielded substantial benefits to the U.S. economy—providing $3 to $17 for every $1 spent over the previous 10 years on implementation of air rules.

Ironically, about 127 million Americans still live in counties where air pollution levels exceed one or more of the six National Ambient Air Standards (NAAQS), and politicians still debate whether the health-based regulation of air quality is hurting the economy and stymieing job growth despite evidence to the contrary. For example, in March 2011, a U.S. Environmental Protection Agency (EPA)-commissioned study ordered by Congress reported that air policy benefits from 1990 to 2020 exceeded $2 trillion, and 160,000 deaths were prevented in just 1 year (U.S. EPA 2011). Another study has estimated that capital investment in air pollution controls on the U.S. power generation sector will create 638,000 construction, installation, and other professional jobs between 2010 and 2015 (Heintz et al. 2011). Clearly, air pollution regulation continues to have important implications for public health and environmental welfare, as well as the health of the economy. To be sure, these critical policy decisions need to be made on a foundation of sound and innovative science.

The question remains: What is left to do regarding air pollution control, and can we further advance our understanding of air pollution risk and find ways to sustainably protect health and the environment? In this regard, a few broad questions do come to mind: What innovative technologies and strategies can be developed, at reasonable cost, to further reduce air pollution in those areas that remain out of compliance or that are subject to local impacts of specific air pollution sources? What new information about how air pollutants affect health is needed to protect those who seem to be at unusual risk (i.e., susceptible)? How important is the mix of air pollutants in the etiology of adverse health outcomes? Finally, given the known interplay between air quality and climate, what will be the impact of climate change on the improvement in air quality that has been achieved to date? These questions are all clearly linked to each other and as we move ahead in the 21st century, real gains in our understanding and improvements in air quality will be achieved only through true interdisciplinary science that can leverage the capabilities and perspectives of the air science community.

In that spirit, in March 2010, the American Association of Aerosol Research (AAAR) held a conference titled “Air Pollution and Health: Bridging the Gap from Sources to Health Outcomes,” which was sponsored by the U.S. EPA and the Health Effects Institute. This conference brought together > 500 scientists from across the air pollution disciplines, along with risk assessors and policy makers, to look at air pollution through a new wide-angle lens. Although great strides had been made in understanding the health impacts of particulate matter (PM) in the past 15 years, uncertainties exist, especially at the lower end of the exposure–response curve. These include the role of PM attributes (composition and size), the spatial and temporal variation of PM, and the significance of these in estimating exposure. Equally important are the extent to which copollutants may interact to alter PM physiochemistry or biological responses, perhaps themselves having poorly appreciated impacts in the multipollutant air environment. With exposure estimates being so critical, consideration was given to atmospheric models of PM and other pollutants that could be wed with health outcomes to improve exposure and risk estimations. Perhaps such a marriage can provide a means to assess whether policy decisions actually have their intended consequence when implemented at such low ambient exposure levels.

As emission sources of air contaminants are fundamental to what ultimately ends up in the air in their emitted forms or as transformed by atmospheric chemistry, the meeting adopted the paradigm of “source to health outcomes” as a logic model to assess the existing state of knowledge, and to establish a foundation for embarking into the multipollutant arena.

A series of technical manuscripts emanated from the conference, some of which are published here in *EHP* and elsewhere. Several overarching messages emerged prominently from the meeting:

- Cross-fertilization among the sciences is essential to further progress in reducing the health assessment uncertainties for PM and other air pollutants, and their real-world combinations. Social science also is important in understanding how people behave both in preventing air pollution and in attempting to avoid its impacts on their lives.
- Regulatory decisions must be soundly based on science. Improved exposure models are needed, as well as an understanding of their connection with ambient air quality models, especially if risks at seemingly low concentrations are to be accurately estimated. These models must be available for local communities (e.g., web accessible) to use in their decisions and planning.
- Monitoring methods have fallen behind technology and need to be upgraded to include multiple pollutants. Monitors need to be judiciously distributed around sources and in densely populated areas. Innovative “personal” monitors—in conjunction with local air quality models—could help communities map out their locales so they can make decisions. These tools would advance exposure science.
- Communities are the keystone to environmental gains, and those at disproportionate exposure or health risk merit special attention. The preponderance of health impacts may lie in these vulnerable populations, and they may be critical to revealing the keys to overall public risk.
- Health impacts, including previously underappreciated outcomes, are being seen at ever lower concentrations, and new mechanisms such as genetic or epigenetic susceptibility are being proposed. We need new biomarkers that reveal both exposure and effect and that also link acute to chronic impacts. These needs are especially true for potentially susceptible groups for which social stress may be contributory.
- Innovative approaches to studying the multipollutant environment can contribute to cost-effective air quality management, especially as transcontinental and transoceanic transport of pollutants come into play. Source-based assessments may help, but we need a better understanding of atmospheric transformation that may result in formation of PM with unclear health impacts. New epidemiological and toxicological tools or strategies are needed.

The 21st century presents a set of new and complex challenges to the air science community, requiring systems approaches and holistic solutions. It is clear that climate change is happening and that there is a significant anthropogenic contribution based on the consensus of 97% of climate scientists. Because air quality and climate are inextricably linked, most sources emit contaminants that impact both the levels of traditional air pollutants and the set of climate “forcers” (carbon dioxide, methane, black carbon, and others) now of great concern. Furthermore, the interrelationship between climate and air quality is highly dynamic. On one hand, air quality is dramatically influenced by climate parameters; for example, higher temperature and altered constituents can increase ozone in some locales. On the other hand, climate-induced changes in the atmosphere, such as increased ozone, are climate forcers. Thus, with the changing landscape of energy options and their varied contributions to the mix of ambient air constituents, it is incumbent that the broad science community approaches the complex issue of air/climate in a holistic manner. Solving 21st-century air pollution and climate problems will require not only heightened awareness of this interplay but also an unprecedented level of cooperation among everyone in the broad science community. Twenty-first century teams need expertise in forecast modeling, adaptation, health and environmental risk assessment, control technologies, and of course the decision and social sciences in developing prevention or intervention strategies

## Figures and Tables

**Figure:**
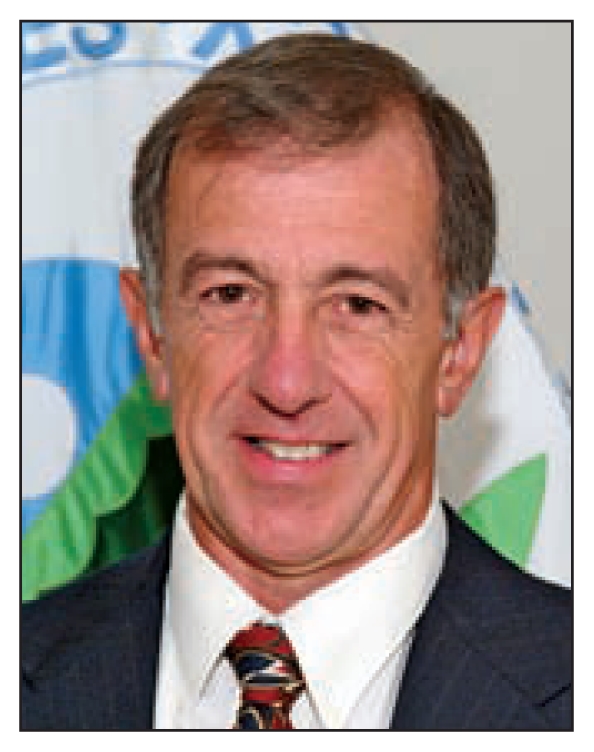
Dan Costa
